# Nur77 attenuates endothelin-1 expression via downregulation of NF-κB and p38 MAPK in A549 cells and in an ARDS rat model

**DOI:** 10.1152/ajplung.00043.2016

**Published:** 2016-10-07

**Authors:** Yujie Jiang, Yi Zeng, Xia Huang, Yueqiu Qin, Weigui Luo, Shulin Xiang, Suren R. Sooranna, Liao Pinhu

**Affiliations:** ^1^The First Clinical Medical College of Jinan University, Guangzhou, Guangdong Province, China;; ^2^Department of Respiratory Medicine;; ^3^Department of Central Laboratory, Youjiang Medical University for Nationalities, Baise, Guangxi, China;; ^4^Department of Digestive, Youjiang Medical University for Nationalities, Baise, Guangxi, China; Youjiang Medical University for Nationalities, Baise, Guangxi, China;; ^5^Department of Intensive Care Unit, the People's Hospital of Guangxi, Nanning, Guangxi, China;; ^6^Department of Surgery and Cancer, Imperial College London, Chelsea and Westminster Hospital, London, United Kingdon; and; ^7^Department of Intensive Care Medicine, Youjiang Medical University for Nationalities, Baise, Guangxi, China

**Keywords:** p38 mitogen-activated protein kinase, nuclear factor-κB, acute respiratory distress syndrome

## Abstract

Acute respiratory distress syndrome (ARDS) is characterized by inflammatory injury to the alveolar and capillary barriers that results in impaired gas exchange and severe acute respiratory failure. Nuclear orphan receptor Nur77 has emerged as a regulator of gene expression in inflammation, and its role in the pathogenesis of ARDS is not clear. The objective of this study is to investigate the potential role of Nur77 and its underlying mechanism in the regulation of endothelin-1 (ET-1) expression in lipopolysaccharide (LPS)-induced A549 cells and an ARDS rat model. We demonstrate that LPS induced Nur77 expression and nuclear export in A549 cells. Overexpression of Nur77 markedly decreased basal and LPS-induced ET-1 expression in A549 cells, whereas knockdown of Nur77 increased the ET-1 expression. LPS-induced phosphorylation and nuclear translocation of NF-κB and p38 MAPK were blocked by Nur77 overexpression and augmented by Nur77 knockdown in A549 cells. In vivo, LPS induced Nur77 expression in lung in ARDS rats. Pharmacological activation of Nur77 by cytosporone B (CsnB) inhibited ET-1 expression in ARDS rats, decreased LPS-induced phosphorylation of NF-κB and p38 MAPK, and relieved lung, liver, and kidney injury. Pharmacological deactivation of Nur77 by 1,1-bis-(3′-indolyl)-1-(p-hydroxyphenyl)methane (DIM-C-pPhOH, C-DIM8) had no effect on ET-1 expression and lung injury. These results indicated that Nur77 decreases ET-1 expression by suppressing NF-κB and p38 MAPK in LPS-stimulated A549 cells in vitro, and, in an LPS-induced ARDS rat model, CsnB reduced ET-1 expression and lung injury in ARDS rats.

acute respiratory distress syndrome (ARDS) is a costly health problem that normally results with high morbidity and mortality (4, 8, 23, 75) and is characterized histologically by diffuse inflammatory alveolar infiltration, hemorrhage, pulmonary edema, and hyaline membrane formation that lead to impaired gas exchange and acute respiratory failure (52, 85). Despite considerable progress in the therapeutic strategies over the past two decades, the mortality rate remains high (10, 82, 83). Better understanding of the pathogenesis in ARDS is necessary to develop effective treatments (25). Increased permeability of the alveolar capillary membrane, which is mediated by excessive inflammatory response initiated in turn by the onset of sepsis, trauma, pneumonia, aspiration, and transfusion, can play a central role in the pathophysiological mechanism of ARDS (52).

Pneumonia is the most common cause for the development of ARDS (79). Lipopolysaccharide (LPS), a major component of the outer membrane of gram-negative bacteria, is the most potent trigger of the innate immunity (60). LPS induces a host of proinflammatory mediator production thought to be involved in the pathogenesis of ARDS, such as TNF-α, IL-6, and IL-8 (63, 80), and is widely used both in vitro and in vivo as experimental approaches to investigate the mechanisms of ARDS.

Endothelin-1 (ET-1), a 21-amino-acid peptide, is predominantly produced in the lungs, where it is expressed in various cell types (17, 21, 34, 49). By binding to its two receptors, ET_A_ and ET_B_, ET-1 exerts its diverse biological activities in the respiratory system, including vasoregulation, bronchoconstriction, cell proliferation, and inflammation, and implicates a wide range of respiratory diseases from asthma to ARDS (18). It is known that systemic and local ET-1 level is elevated in clinical and experimental ARDS, which correlated with disease severity and poor clinical outcomes (1, 29, 58, 62). ET-1 causes endothelial and epithelial dysfunction in ARDS by inducing proinflammatory mechanisms and cytokine secretion (15, 26, 28), and administration of an endothelin receptor antagonist modulates the extent of lung injury, suggesting a promising therapeutic target (67, 98). Elucidating the molecular mechanisms of ET-1 induction can lead to potential new treatments (34). Compared with the endothelium, less is known about the mechanisms of alveolar epithelial dysfunction, which is pivotal in alveolar flooding and leukocyte accumulation (51).

Nur77, a member of the nuclear hormone receptor 4A (NR4A) subgroup of the nuclear receptor superfamily, is encoded by an immediate early gene and expressed in multiple tissues in response to diverse stimuli (69). Originally, Nur77 was described as an inducer of apoptosis in thymocyte selection (38, 87, 88), but it has now been shown to regulate cell proliferation, differentiation, and survival. As a transcription factor, Nur77 targets genes involved in metabolism, cancer, immunity, atherosclerosis, and inflammatory diseases (35, 57). In vitro studies showed a rapid induction of Nur77 expression in macrophages and monocytes after LPS treatment or other inflammatory stimuli (70), and pharmacological activation or overexpression of Nur77 resulted in downregulation of inflammatory cytokines and chemokines, such as IL-1β, IL-6, and IL-8 (71). In vivo, Nur77 is expressed aberrantly in inflammatory diseases, such as lung cancer, atherosclerotic lesions, multiple sclerosis, and inflamed human synovial tissue (2, 9, 35), and Nur77 knockout mice are more susceptible to a variety of diseases, including sepsis, airway inflammatory diseases, atherosclerosis, colitis, inflammatory bowel diseases, and cerebral ischemia, all of which can be attributed to an enhanced inflammatory response (24, 36, 40, 41). These data indicated the anti-inflammatory effects of Nur77 both in vitro and in vivo. However, its role in the pathogenesis of ARDS has yet to be fully investigated.

## MATERIALS AND METHODS

### 

#### Cell culture.

A549 cells were obtained from American Type Culture Collection (VR-15; ATCC). Cells were cultured in RPMI-1640 medium containing 10% (vol/vol) fetal bovine serum (GIBCO) at 37°C in a 5% CO_2_ incubator. All studies on these cells were performed with subcultures after three to five passages.

#### Nur77 overexpression and knockdown in A549 cells.

Human full-length Nur77 (exNur77) was constructed in pcDNA3.1 plasmid vector, and the empty vector served as a negative control (exNC). Short-hairpin RNA targeting human Nur77 [shNur77, 5′-**GCCAAACTGGACTACTCCAAGT**TTCAAGAGAACTTGGAGTAGTCCAGTTTGGTT-3′ (bold sequences are target sequences; underlined sequences represent hairpin)] was constructed in pGPU6/Neo plasmid vector, and a plasmid carrying a nontargeting control sequence served as a negative control [shNC, 5′-**GTTCTCCGAACGTGTCACGT**CAAGAGATTACGTGACACGTTCGGAGAATT-3′ (bold sequences are non-targeting sequences; underlined sequences represent hairpin)]. All of the plasmids mentioned above were synthesized by GenePharma (Shanghai, China). A549 cells at ∼60–80% confluence were transfected using Lipofectamine 2000 Reagent (11668-027; Life Technologies, Carlsbad, CA) according to the manufacturer's instructions, and stable cell lines were obtained by geneticin 418 (11811-023; Invitrogen) screening. Overexpression and knockdown of Nur77 were verified by real-time PCR (RT-PCR).

#### Cell viability assay.

Cell viability was assessed using a commercially available MTT Assay Kit (AR1156; Boster, Wuhan, China) according to the manufacturer's instructions. The formazan product was dissolved in dimethyl sulfide, and the optical density was measured at 570 nm using a colorimetric microplate reader (BioTek, Winooski, VT).

#### Cell treatment and sample collection.

The cells were divided into 12 groups as follows: control group (CTL, cells without LPS treatment); LPS group [LPS, LPS treatment at 10 μg/ml (*Escherichia coli*, serotype 055:B5, L2880; Sigma-Aldrich)]; LPS and SB-203580 group [LPS + SB, the cells were preincubated with 10 μM SB-203580 (13) (S8307; Sigma, St. Louis, MO) for 1 h following LPS treatment]; LPS and SC-514 group [LPS + SC, the cells were preincubated with 10 μM SC-514 (45) (SML0557; Sigma) for 1 h following LPS treatment]; shNur77 group (shNur77, the Nur77 knockdown cells without LPS treatment); shNur77 + LPS group (shNur77 + LPS, the Nur77 knockdown cells with LPS treatment at 10 μg/ml); shNC group (shNC, the cells transfected with shNC without LPS treatment); shNC + LPS group (shNC + LPS, the cells transfected with shNC were treated with LPS at 10 μg/ml); exNur77 group (exNur77, the Nur77 overexpression cells without LPS treatment); exNur77 + LPS group (exNur77 + LPS, the Nur77 overexpression cells with LPS treatment at 10 μg/ml); exNC group (exNC, the cells transfected with exNC without LPS treatment); and exNC + LPS group (exNC, the cells transfected with exNC were treated with LPS at 10 μg/ml).

The culture supernatant was harvested 4 h after LPS stimulation for enzyme-linked immunosorbent assay (ELISA) measurement of ET-1 (DET100; Sigma). RNA was isolated from the cells 4 h after LPS stimulation by using the RNeasy Mini Kit (74104; Qiagen). RNA integrity was checked electrophoretically and quantified by using spectrophotometry. Cell lysates were collected at 15 min and at 4 h after LPS stimulation by using RIPA buffer (89900; Thermo Scientific) containing 25 mM Tris·HCl, pH 7.6, 150 mM NaCl, 1% sodium deoxycholate, 1% Nonidet P-40, 0.1% SDS, and proteinase inhibitor cocktail containing 10 μg/ml leupeptin, 20 μg/ml aprotinin, and 2 mM phenylmethylsulfonyl fluoride (PMSF) following the manufacturer's protocol for Western blotting analysis of phospho-NF-κB p65 and phospho-p38 MAPK, and Nur77, respectively. Cytoplasmic fractions were extracted, and coimmunoprecipitation (co-IP) was performed 2 h after LPS challenge using the Universal Magnetic Co-IP kit (54002; Active Motif) according to the manufacturer's instructions. Briefly, A549 cells were allowed to swell in hypotonic buffer containing phosphatase inhibitors, deacetylase inhibitor, protease inhibitor cocktail, and PMSF and then lysed in 5% detergent. Lysates were subsequently centrifuged for 30 s at 14,000 *g* at 4°C. The supernatant was collected and processed to co-IP. For immunofluorescence analysis of subcellular location of phospho-NF-κB p65 and phospho-p38 MAPK by confocal laser scanning microscopy, the cells were fixed in 4% paraformaldehyde 30 min after LPS treatment; for immunofluorescence analysis of Nur77, the cells were fixed 2 h after LPS treatment. The treatment strategy is summarized in Table 1.

**Table 1.** Representation of the timeline of animal experiments

Timeline				Procedure
−1 h	−1/2 h	0 h	2–24 h	
			Blood, BALF, lung, liver, and kidney tissue	CTL
		LPS (10 mg/kg iv)	Blood, BALF, lung, liver, and kidney tissue	LPS
	SB-203580 (5 mg/kg, iv)	LPS (10 mg/kg iv)	Blood, BALF, lung, liver, and kidney tissue	LPS + SB-203580
	SC-514 (5 mg/kg, iv)	LPS (10 mg/kg iv)	Blood, BALF, lung, liver, and kidney tissue	LPS + SC-514
C-DIM8 (20 mg/kg orally)		LPS (10 mg/kg iv)	Blood, BALF, lung, liver, and kidney tissue	LPS + DIM8
CsnB (25 mg/kg iv)		LPS (10 mg/kg iv)	Blood, BALF, lung, liver, and kidney tissue	LPS + CsnB

Data for −1, −1/2, and 0 h indicate treatment conditions. Data for 2–24 h indicate samples collected 2, 4, 6, and 24 h after death. BALF, bronchoalveolar lavage; CTL, control; LPS, lipopolysaccharide; DIM8, 1,1-bis-(3′-indolyl)-1-(p-hydroxyphenyl)methane; CsnB, cytosporone B.

#### Animals and experimental protocol.

Male specific pathogen-free rats of Sprague-Dawley strain weighing 200–250 g (6–8 wk old) were obtained from the Laboratory Animal Center of Youjiang Medical University for Nationalities (Baise, Guangxi Province, China). The study was approved by the Committee of Animal Care and Use of Youjiang Medical University for Nationalities, and all procedures were performed according to the National Institutes of Health Guidelines. The ARDS rat model was induced by intravenous injection of LPS as described previously (54). The rats were housed individually at a constant temperature (22 ± 2°C) and humidity with a 12:12-h light-dark cycle and free access to chow and water. The rats were randomized into the following six groups: control group (CTL, *n* = 24, treated with caudal vein injection of saline); LPS group (LPS, *n* = 24, induced by caudal vein injection of 5 mg/kg LPS); LPS and SC-514 group [LPS + SC, *n* = 24, pretreated with caudal vein injection of 5 mg/kg SC-514 (45) for 30 min following 5 mg/kg LPS injection iv]; LPS and SB-203580 group [LPS + SB, *n* = 24, pretreated with caudal vein injection of 5 mg/kg SB-203580 for 30 min (46) following 5 mg/kg LPS injection iv]; LPS + CsnB group [CsnB, pretreated with caudal vein injection of 25 mg/kg cytosporone B (40) (CsnB, sc-252653; Santa Cruz, Santa Cruz, CA) for 1 h following 5 mg/kg LPS injection iv]; LPS + C-DIM8 group [C-DIM8; pretreated with oral gavage of 20 mg/kg (16) DIM-C-pPhOCH (DIM-8; synthesized by SKS Chem) for 1 h following 5 mg/kg iv injection of LPS]. The treatment strategy is summarized in Table 2.

**Table 2.** Representation of the timeline of cell experiments

Timeline		Group
−1 h	0 h	
	PBS	CTL
	LPS (10 μg/ml)	LPS
	PBS	shNur77
	LPS (10 μg/ml)	shNur77 + LPS
	PBS	shNC
	LPS (10 μg/ml)	shNC + LPS
	PBS	exNur77
	LPS (10 μg/ml)	exNur77 + LPS
	PBS	exNC
	LPS (10 μg/ml)	exNur + LPS
SB-203580 (10 μM)	LPS (10 μg/ml)	LPS + SB-203580
SC-514 (10 μM)	LPS (10 μg/ml)	LPS + SC-514

Timeline data indicate treatment conditions. shNur77, short-hairpin RNA targeting human Nur77; shNC, plasmid carrying a nontargeting control sequence served as a negative control; exNur77, human full-length Nur77; exNC, empty vector serving as a negative control.

After anesthesia with chloral hydrate (400 mg/kg ip), blood gases and creatinine were determined by the i-STAT portable analyzer (i-STAT 300; Abbott Laboratories, Abbott Park, IL), and then blood samples, bronchoalveolar lavage fluid (BALF), lung tissues, liver tissues, and kidney tissues were collected before at 2, 4, 6, and 24 h (*n* = 6 for each time point) after LPS injection. The blood samples were collected into serum separator tubes, allowed to clot for 30 min before centrifugation at 2,000 *g* for 20 min, separated into aliquots, and stored at −80°C for analysis. After thoracotomy and laparotomy were performed, the right main bronchus was tied with a string at the right hilum, and BALF was obtained by cannulating the left main bronchus with a 21-gauge catheter and then lavaging the left lung four times with 3 ml of PBS. BALF recovery was always >85%. The obtained BALF was centrifuged at 1,000 *g* for 10 min at 4°C, and the cell-free supernatant was analyzed for total protein and myeloperoxidase (MPO). The cell pellets were resuspended in 50 μl of PBS, and the total cell number was calculated using a hemocytometer. Cytospins of BALF were prepared and stained with the Wright-Giemsa Stain Kit (D010; Jiancheng, Nanjing, China). Neutrophils were identified using light microscopy by their characteristic nuclei, and the neutrophil percentage in the BALF was determined by counting a total of 500 cells. The total number of neutrophils was calculated as the percentage of neutrophils multiplied by the total cell numbers in the recovered BALF. The protein concentration in BALF was measured using a BCA protein assay kit (Beyotime, Shanghai, China). The upper lobe of right lung, liver, and kidney were fixed for histopathological and immunohistochemical examination, the middle lobe of right lung was used for measurement of lung wet-to-dry weight ratios, and the remaining lower lobe of right lung was stored at −80°C and used to isolate total RNA and/or protein. Total RNA of lung tissue was extracted by phenol/chloroform using TRIzol LS Reagent (10296-010; Invitrogen) according to the instruction of the manufacturer. For histopathological and immunohistochemical staining, the lung, liver, and kidney were fixed in 10% neutral formaldehyde, embedded in paraffin wax, and sectioned (4 μm thickness) and stained with hematoxylin and eosin (HE) or subjected to immunohistochemistry. Lung tissues were homogenized with 10 vol of 20 mM Tris·HCl (pH 7.4), 250 mM NaCl, 1 mM Na_3_VO_4_, 3 mM EDTA, 2 mM dithiothreitol, 3 mM EGTA, 20 mM glycerophosphate, 0.6% Nonidet P-40, 0.5 mM PMSF, 60 μg/ml aprotinin, and 1 μg/ml leupeptin on ice using a homogenizer. The homogenate was gently rotated for 30 min at 4°C followed by centrifugation at 13,000 *g* for 10 min at 4°C, and the supernatants were removed and stored at −80°C for ELISA and Western blotting analysis.

#### Quantitative RT-PCR.

The expression of ET-1 and Nur77 mRNA of A549 cells and rat lung tissue was analyzed by RT-PCR. Strand DNA was synthesized with the RevertAid First Strand cDNA Synthesis Kit (K1622; Thermo Scientific) as per the manufacturer's instructions. The quantitative RT-PCR was performed on an iQ5 multicolor RT-PCR detection system (Bio-Rad) by using FastStart Universal SYBR Green Master (Rox, 10356100; Roche) according to the manufacturer's instructions. The primers for the amplification of Nur77, ET-1, and GAPDH [GenBank accession no. NM_173157.2 (Nur77 human)/NM_024388.2 (Nur77 rat)/NM_001955.4 (ET-1 human)/NM_012548 (ET-1 rat)/NM_001289746 (GAPDH human)/NM_017008 (GAPDH rat)] were designed using primer premier 5 software (Premier Biosoft) and synthesized by Shenggong (Shanghai, China). The primer sequences were as follows: Nur77 (human) sense: 5′-ATACACCCGTGACCTCAACCA-3′, antisense: 5′-TTCTGCACTGTGCGCTTGAA-3′; Nur77 (rat) sense: 5′-CGTGCCTTTAAGCCCATAGC-3′, antisense: 5′-TCTGGAATGAGGAGATACATCAGTCT-3′; ET-1 (human) sense: 5′-AAACCCACTCCCAGTCCACC-3′, antisense: 5′-CCAAGTCCATACGGAACAACG-3′; ET-1 (rat) sense: 5′-CTGGACATCATCTGGGTCAACA-3′, antisense: 5′-GGCTCGGAGTTCTTTGTCTGC-3′; GAPDH (human) sense: 5′-ccacccatggcaaattccatggca-3′, antisense: 5′-tctagacggcaggtcaggtccacc-3′; and GAPDH (rat): sense: 5′-CGTATCGGACGCCTGGTTA-3′, antisense: 5′-GACTGTGCCGTTGAACTTGC-3′.

After 5 min of initial denaturation at 95°C, PCR was carried out for 40 cycles at 95°C for 10 s and 60°C for 30 s. GAPDH was performed simultaneously and used as the housekeeping gene. The threshold cycle (C_t_) value was measured, and the comparative gene expression was calculated by the 2^−ΔΔC_t_^ method as described previously.

#### Enzyme-linked immunosorbent assay.

ET-1 concentration in cell culture supernatants, rat sera, and rat lung tissue homogenates was determined by using a Quantikine ELISA kit (DET100; R&D Systems), following the manufacturer's protocol. MPO concentrations in the BAL and lung tissue were measured with a rat MPO ELISA kit (HK105; Hycult Biotech, Plymouth Meeting, PA) according to the manufacturer's instructions.

#### Western blotting.

Protein concentrations of rat lung tissue homogenate and cell lysates were measured using the bicinchoninic acid protein assay kit (Beyotime) with bovine serum albumin (BSA) as the standard. Extracts containing equal amounts of total protein (20 μg) were loaded on SDS-PAGE (4–15% polyacrylamide), subjected to electrophoresis, and electrophoretically transferred to a polyvinylidene difluoride filter membrane that was then blocked for 2.5 h at room temperature with 5% nonfat milk in PBS (137 mM NaCl, 8.1 mM Na_2_HPO_4_, 2.7 mM KCl, and 1.5 mM KH_2_PO_4_) containing 0.1% Tween 20. The membrane was washed three times with PBS-Tween buffer, incubated with primary antibodies against phospho-NF-κB p65 (1:1,000 dilution, 3033; Cell Signaling Technology, Danvers, MA), phospho-p38 MAPK (1:1,000 dilution, 4511; Cell Signaling Technology), Nur77 (1:1,000 dilution, sc-5569; Santa Cruz), and β-actin (1:2,000 dilution, sc-47778; Santa Cruz) in PBS-Tween buffer at 4°C overnight, and rinsed three times with PBS-Tween buffer, and then the membrane was incubated with horseradish peroxidase-conjugated goat anti-rabbit IgG (1:5,000 dilution) for 60 min at room temperature. The blots were subsequently washed three times in PBS-Tween buffer followed by visualization with enhanced chemiluminescence reagents (AR1171; Boster) and exposed to an X-ray film (Fuji Photo Film, Tokyo, Japan). The scanned images were imported into an image analyzer (ImageJ version 1.48; National Institutes of Health, Bethesda, MD). Scanning densitometry was used for semiquantitative analysis of the data.

#### Co-IP assay.

Co-IP were performed using the Universal Magnetic Co-IP kit (54002; Active Motif) as per the manufacturer's instructions. In brief, cytoplasmic extracts (500 μg) were incubated with anti-Nur77 (5 μg, sc-166166; Santa Cruz) or negative control IgG (5 μg, sc-2025; Santa Cruz) at 4°C on a rotator for 4 h. After a short centrifugation, protein complexes were incubated with 25 μl protein G magnetic beads for 1 h at 4°C on a rotator. Subsequently, the protein beads were precipitated using a magnet. Immune complexes were collected in 20 μl 2× reducing loading buffer (130 mM Tris, pH 6.8, 4% SDS, 0.02% bromphenol blue, 20% glycerol, 100 mM DTT) after four washes with Co-IP/wash buffer and then subjected to Western blotting with anti-Nur77 (sc-5569; Santa Cruz), anti-NF-κB p65 (8242; Cell Signaling Technology), and anti-p38 MAPK (8690; Cell Signaling Technology).

#### Immunofluorescence analysis by confocal laser scanning microscopy.

A549 cells were seeded (5 × 10^4^/ml) on glass bottom cell culture dishes (NEST, Wuxi, China), incubated overnight, and then treated as described previously. Cells were rinsed two times with 1× PBS (pH 7.4) and fixed in 4% paraformaldehyde at room temperature for 30 min. Fixed cells were washed three times and then permeabilized with 1 ml of 0.3% Triton X-100 for 20 min. After blocking with PBS containing 4% BSA for 30 min, cells were incubated with primary antibody (phospho-p38 MAPK, 1:50 dilution; phospho-NF-κB p65, 1:50 dilution; and Nur77, 1:50 dilution) in 4% BSA/PBS overnight at 4°C and then washed and incubated with FITC-conjugated anti-rabbit IgG antibody (1:150 dilution) for 1 h at room temperature. Cells were washed, dried, and mounted in medium containing DAPI (AR1176; Boster) and imaged on an Olympus Fluoview 500 IX71 Confocal Laser Scanning Microscope.

#### Histological analysis.

The right upper lobe of the lung, liver, and kidney were fixed in 10% neutral formaldehyde at 4°C overnight, embedded in paraffin wax, and sectioned (4 μm) for HE staining. Six slices were selected from each group, and six fields of each slice were visualized by an Olympus BX53F microscope (Olympus, Tokyo, Japan) with an Olympus DP73 digital camera (Olympus). Pathologists unaware and blinded to the nature and characteristics of the sample examined the HE-stained slides. The degree of pathological damage was evaluated based on lung the injury scoring system (53), which was assessed on a scale of 0–2 for each of the following criteria: *1*) neutrophils in the alveolar space, *2*) neutrophils in the interstitial space, *3*) number of hyaline membranes, *4*) amount of protein-associated debris, and *5*) extent of alveolar septal thickening. The total injury score was derived from the following calculation: score = [20 × (1) + 14 × (2) + 7 × (3) + 7 × (4) + 2 × (5)]/(no. of fields × 100). Liver injury was defined as the amount of destruction of hepatic lobules, hemorrhage, infiltration of inflammatory cells, and hepatocyte necrosis observed and scored from 1 through 4 according to percentage of area of involvement per high-power field. Kidney tubular damage was defined as tubular epithelial swelling, vacuolar degeneration, necrotic tubules, loss of brush border, cast formation and desquamation observed and scored from 1 through 4 according to the percentage of area of involvement per high-power field.

Immunohistochemistry was performed after blocking endogenous peroxidase activity with 3% H_2_O_2_ and methanol for 10 min and nonspecific protein binding with 10% sheep serum for 15 min. Sections were then incubated with ET-1 antibody (1:400 dilution, ab2786; Abcam, Cambridge, MA) or Nur77 (1:200 dilution, sc-5569; Santa Cruz) overnight at 4°C. After three washes of 5 min with PBS, sections were incubated with peroxidase-conjugated IgG antibody. Following three rinses of 5 min with PBS, the slides were then stained using the biotin-avidin peroxidase method. After development, slides were counterstained with hematoxylin.

#### Statistical analysis.

Data normality was determined by Shapiro-Wilk and Kolmogorov-Smirnov tests (α = 0.05). The data with normality distribution are presented as means ± SE and were analyzed using the two-tailed Student's *t*-test for comparison between two groups or one-way analysis of variance followed by Bonferroni's post hoc test for multiple comparisons. Histological scores were analyzed with the Kruskal-Wallis nonparametric test followed by Dunn's pairwise comparison. Significance was defined as *P* values <0.05. All tests were performed using SPSS version 21.0 software (IBM-SPSS, Chicago, IL).

## RESULTS

### 

#### LPS induces Nur77 mRNA and protein expression and nuclear export in A549 cells.

**Fig. 1.**
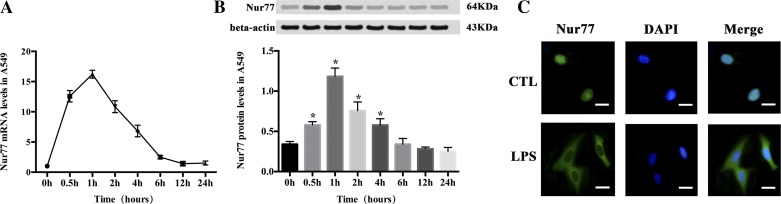
Lipopolysaccharide (LPS) exposure induces Nur77 expression and nuclear export in A549 cells. An induction of Nur77 mRNA (*A*) and protein expression (*B*) was observed after LPS (10 μg/ml) stimulation. Nur77 mRNA expression was detected by quantitative real-time PCR (RT-PCR), and Nur77 protein expression was determined by Western blotting analysis after A549 cells were treated with LPS (10 μg/ml) for the indicated times. Data are presented as means ± SE of 3 independent experiments performed in triplicate. *P < 0.05 vs. 0 h. *C*: Nur77 nuclear export was detected after LPS stimulation. The subcellular location of Nur77 was examined by immunostaining using anti-Nur77 antibody and observed by confocal microscopy at 15, 30, and 60 min and 2, 6, and 12 h. Nur77 nuclear export was detectable at 30 min, which appears to be irreversible during the observed duration. About 90% of control cells exhibited nuclear staining of Nur77, whereas ∼80% of LPS-stimulated cells displayed cytoplasmic distribution of Nur77. The original magnification is ×400. Bars, 10 μm.

Nur77 mRNA and protein expression increased after LPS stimulation (Fig. 1, *A* and *B*), and Nur77 was shown to be transferred from nuclei to cytoplasm (Fig. 1*C*).

#### Nur77 inhibits basal and LPS-induced ET-1 production through suppression of activation of NF-κB p65 and p38 MAPK in A549 cells.

**Fig. 2.**
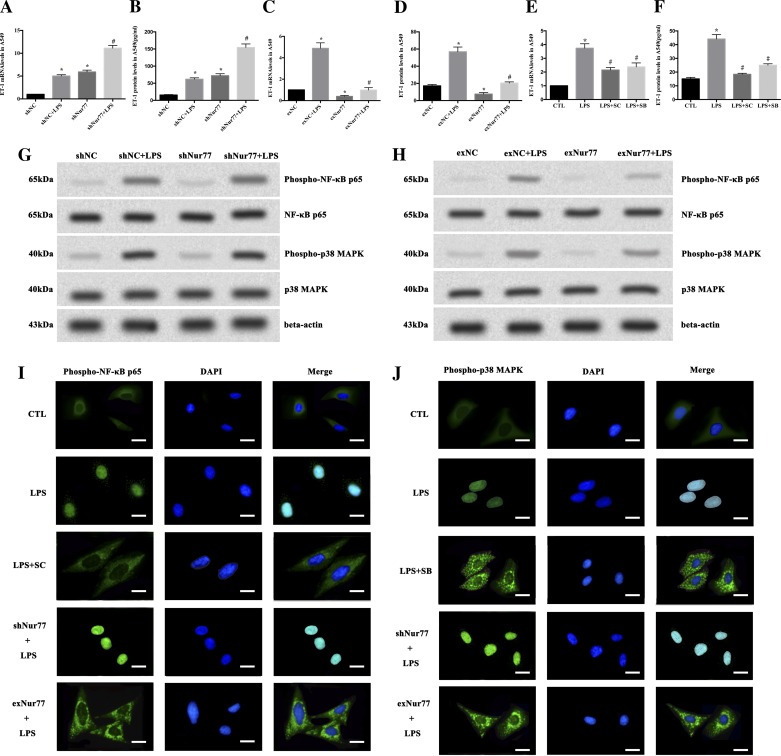
Nur77 inhibits basal and LPS-induced endothelin-1 (ET-1) expression in A549 cells by suppression of NF-κB and p38 MAPK activation in A549 cells. Nur77 knockdown augments both basal and LPS-induced ET-1 expression in A549 cells (*A* and *B*). A549 cells transfected with short-hairpin RNA targeting human Nur77 (shNur77) or a plasmid carrying a nontargeting control sequence (shNC) were incubated with or without LPS (10 μg/ml) for 4 h. The expression of ET-1 mRNA (*A*) was detected by quantitative RT-PCR (qRT-PCR), and ET-1 protein (*B*) was determined by ELISA. *P < 0.05 vs. shNC. #*P* < 0.05 vs. shNC + LPS. Data are presented as means ± SE of 3 independent experiments performed in triplicate. Nur77 overexpression decreases both basal and LPS-induced ET-1 expression in A549 cells (*C* and *D*). A549 cells transfected with pcDNA3.1-Nur77 (exNur77) or empty vector (exNC) were incubated with or without LPS (10 μg/ml) for 4 h. The expression of ET-1 mRNA was detected by qRT-PCR (*C*), and ET-1 protein was determined by ELISA (*D*). *P < 0.05 vs. exNC. #*P* < 0.05 vs. exNC + LPS. Data are presented as means ± SE of 3 independent experiments performed in triplicate. SC-514 and SB-203580 inhibit LPS-mediated ET-1 mRNA and protein expression in A549 cells (*E* and *F*). The mRNA expression of ET-1 was measured 4 h after LPS stimulation by qRT-PCR (*E*). The supernatant ET-1 levels were determined 24 h after LPS challenge using ELISA (*F*). CTL, A549 cells incubated with medium alone; LPS, A549 cells stimulated with LPS (10 μg/ml); LPS + SC, cells preincubated with SC-514 (10 μM) for 1 h before stimulation with LPS; LPS + SB, cells preincubated with SB-203580 (10 μM) for 1 h before LPS administration. *P < 0.05 vs. CTL. #*P* < 0.001 vs. LPS. Data are presented as means ± SE of 3 independent experiments performed in triplicate. Knockdown of Nur77 augments LPS-induced phosphorylation of NF-κB p65 and p38 MAPK in A549 cells(*G*). A549 cells transfected with shNur77 or shNC were incubated with LPS (10 μg/ml) for 15 min, and activation of NF-κB p65 and p38 MAPK was determined by Western blotting with corresponding antibodies. β-Actin served as loading control. Representative results of 3 independent experiments performed in triplicate are shown. Overexpression of Nur77 decreases LPS-induced phosphorylation of NF-κB p65 and p38 MAPK in A549 cells (*H*). A549 cells transfected with pcDNA3. 1-Nur77 (exNur77) or empty vector (exNC) were incubated with LPS (10 μg/ml) for 15 min, and activation of NF-κB p65 and p38 MAPK was determined by Western blotting with corresponding phospho-specific antibodies. β-Actin was employed as loading control. These blots are representative of 3 independent experiments performed in triplicate. Nur77 inhibits LPS-induced phospho-NF-κB p65 and phospho-p38 MAPK nuclear translocation in A549 cells (*I* and *J*). LPS-induced phospho-NF-κB p65 nuclear translocation was blocked by Nur77 overexpression and was increased by Nur77 knockdown (*I*). Bars, 10 μm. LPS-induced phospho-p38 MAPK nuclear translocation was blocked by Nur77 overexpression and was increased by Nur77 knockdown (*J*). Subcellular distribution of phospho-NF-κB p65 and phospho-p38 MAPK was visualized by laser scanning confocal microscopy with corresponding specific antibodies (green) 30 min after LPS challenge. Nuclei were counterstained with DAPI (blue). Bars, 10 μm.

ET-1 mRNA and protein expression was augmented in Nur77 knockdown A549 cells at both basal and LPS-stimulated conditions (Fig. 2, *A* and *B*). Overexpression of Nur77 decreased basal and LPS-induced ET-1 mRNA and protein expression (Fig. 2, *C* and *D*). Both SC-514 and SB-203580 inhibited LPS-induced ET-1 mRNA and protein expression (Fig. 2, *E* and *F*).

LPS-induced phosphorylation of NF-κB p65 and p38 MAPK was enhanced by knockdown of Nur77 (Fig. 2*G*) and was suppressed by overexpression of Nur77 (Fig. 2*H*). SC-514 and SB-203580 inhibited LPS-induced phosphorylation of NF-κB p65 and p38 MAPK, respectively (data not shown).

LPS triggered phospho-NF-κB p65 and phospho-p38 MAPK to transfer from cytoplasm to nuclei (Fig. 2, *I* and *J*, *top*). Nur77 overexpression blocked this nuclear translocation, and Nur77 knockdown promoted it (Fig. 2, *I* and *J*, *bottom*). The LPS-induced redistribution of phospho-NF-κB p65 and phospho-p38 MAPK was partially inhibited by pretreatment with SC-514 and SB-203580, respectively (Fig. 2, *I* and *J*, *middle*).

#### Cytoplasmic Nur77 interacts with NF-κB p65 and p38 MAPK in LPS-induced A549 cells.

**Fig. 3.**
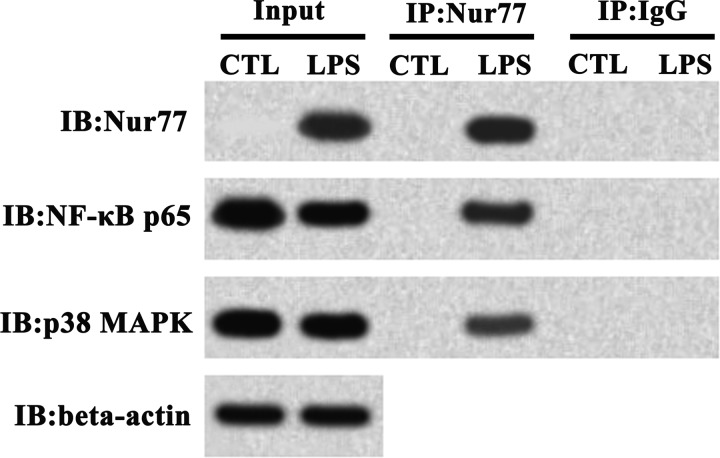
Endogenous Nur77 coimmunoprecipitates NF-κB p65 and p38 MAPK in A549 cells. Cytoplasmic extracts prepared 2 h after LPS administration were immunoprecipitated with anti-Nur77 or control IgG antibody, and immunoprecipitates and the input (10% of the cytoplasmic extracts) were subjected to immunoblotting analysis with anti-NF-κB p65, anti-p38 MAPK, and anti-Nur77. β-Actin was used as loading control. A representative image of 3 independent experiments performed in triplicate is shown. IP, immunoprecipitation; IB, immunoblot.

Co-IP was performed to test the interactions between NF-κB p65/p38 MAPK and Nur77 with cytoplasmic extracts of A549 cells with or without LPS treatment. As shown in Fig. 3, immunoprecipitation of Nur77 pulled down both NF-κB p65 and p38 MAPK, indicating that both NF-κB p65 and p38 MAPK interact with Nur77 in LPS-stimulated A549 cells.

#### CsnB inhibits LPS-induced ET-1 expression through induction of Nur77 in ARDS rats.

**Fig. 4.**
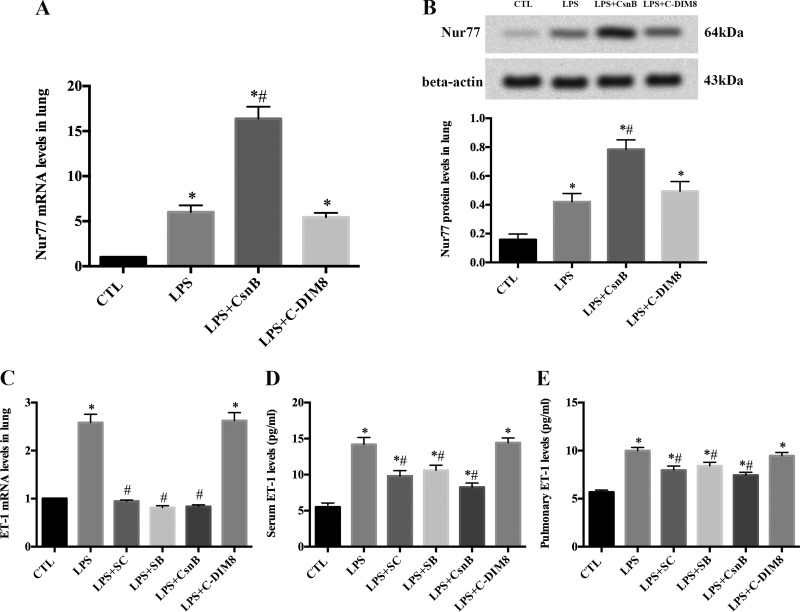
Cytosporone B (CsnB) inhibits LPS-induced ET-1 production by activation of Nur77 in acute respiratory distress syndrome (ARDS) rats. Pretreatment with CsnB increased LPS-induced Nur77 mRNA and protein expression in lung. Pretreatment with 1,1-bis-(3′-indolyl)-1-(p-hydroxyphenyl)methane (C-DIM8) did not influence Nur77 expression. Nur77 mRNA (*A*) and protein expression (*B*) were detected with qRT-PCR and Western blotting, respectively, 4 h after LPS stimulation. *P < 0.05 vs. CTL. #*P* < 0.05 vs. LPS. CsnB, SC-514, and SB-203580 inhibited LPS-induced ET-1 mRNA expression in lung; C-DIM8 failed to regulate ET-1 mRNA expression. ET-1 mRNA was measured by qRT-PCR 4 h after LPS administration. *P < 0.05 vs. CTL. #*P* < 0.05 vs. LPS (*C*). CsnB, SC-514, and SB-203580 inhibited LPS-induced serum and pulmonary ET-1 protein levels; C-DIM8 did not influence serum and pulmonary ET-1 levels. Serum (*D*) and pulmonary (*E*) ET-1 levels were determined by ELISA at 4 h after LPS administration. Data are presented as means ± SE of 3 independent experiments performed in triplicate. *P < 0.05 vs. CTL. #*P* < 0.05 vs. LPS.

CsnB, an agonist for Nur77, increased Nur77 mRNA and protein expression in LPS-induced ARDS rats (Fig. 4, *A* and *B*), whereas C-DIM8 did not change Nur77 expression in LPS-induced ARDS rats (Fig. 4, *A* and *B*). CsnB as well as SC-514 and SB-203580 inhibited ET-1 mRNA expression in lung (Fig. 4*C*) and ET-1 protein levels in serum and lung (Fig. 4, *D* and *E*) in ARDS rats.

#### CsnB suppressed LPS-induced NF-κB and p38 MAPK activation in ARDS rats.

**Fig. 5.**
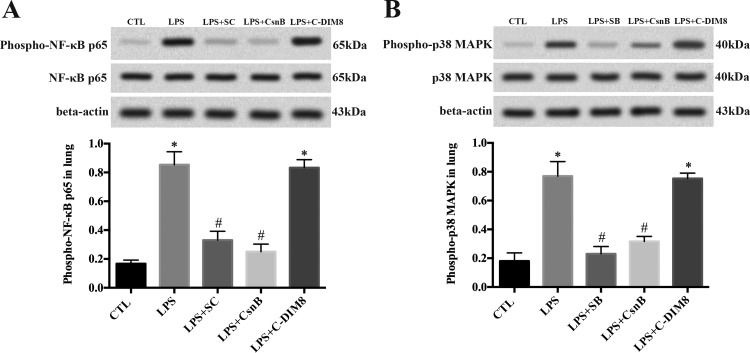
CsnB inhibits LPS-induced NF-κB and p38 MAPK activation in ARDS rats. CsnB and SC-514 suppressed LPS-induced phosphorylation of NF-κB p65 in ARDS rats (*A*). CsnB and SB-203580 suppressed LPS-induced phosphorylation of p38 MAPK in ARDS rats (*B*). Phosphorylation of NF-κB p65 and p38 MAPK was determined by Western blot with corresponding phospho-specific antibodies 2 h after LPS stimulation. β-Actin served as loading control. Data are presented as means ± SE of 3 independent experiments performed in triplicate. *P < 0.05 vs. CTL. #*P* < 0.05 vs. LPS.

LPS activated NF-κB and p38 MAPK signaling by increased phosphorylation of NF-κB p65 and p38 MAPK, whereas pretreatment with CsnB partly blocked LPS-induced phosphorylation of NF-κB p65 and p38 MAPK (Fig. 5, *A* and *B*). SC-514 and SB-203580 pretreatment also exhibited similar effect on LPS-induced phosphorylation of NF-κB p65 and p38 MAPK, respectively (Fig. 5, *A* and *B*).

#### CsnB inhibits LPS-induced ET-1 expression in lung by activation of Nur77 in ARDS rats.

**Fig. 6.**
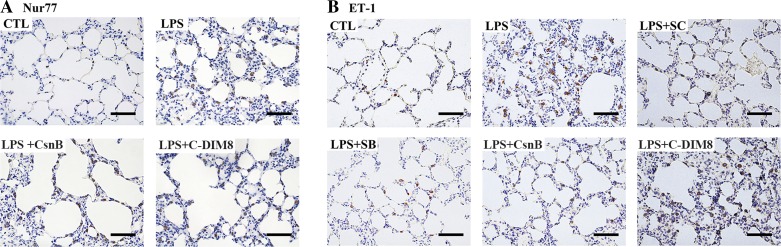
CsnB inhibits LPS-induced ET-1 expression in lung by activation of Nur77 in ARDS rats. Immunohistochemistry was performed to identify Nur77 subcellular localization and protein expression, as well as ET-1 protein expression, in lung tissue 24 h after LPS administration. CsnB enhanced LPS-induced cytoplasmic translocation and protein expression of Nur77 in lung tissue in ARDS rats. C-DIM8 did not affect Nur77 expression in lung in ARDS rats. Nur77 protein (dark brown) was stained with anti-Nur77 antibody, and the nuclei (blue) were stained with hematoxylin. Mild nuclear Nur77 expression was observed in alveolar cells in lung tissue of the CTL group. LPS administration resulted in strong cytoplasmic Nur77 expression, and CsnB pretreatment further enhanced it (*A*). CsnB inhibited LPS-induced ET-1 expression in lung tissue in ARDS rats. ET-1 protein (dark brown) was stained with anti-ET-1 antibody, and nuclei (blue) were counterstained with hematoxylin. Mild ET-1 expression was observed in the cytoplasm of alveolar cells in lung tissue of the CTL group. However, LPS treatment resulted in increased ET-1 expression in lung. Pretreatment with CsnB, SC-514, and SB-203580 decreased LPS-induced ET-1 protein expression. C-DIM8 did not affect ET-1 protein expression in lung (*B*). Images are representative of 6 rats/group in 2 separate experiments. Bars, 50 μm.

Immunohistochemistry of lung tissue sections shows that Nur77 is expressed in alveolar epithelial cells, airway epithelial cells, alveolar macrophages, and bronchial smooth muscle (Fig. 6*A*) and exhibited a predominantly nuclear staining pattern in the CTL group. Treatment with LPS resulted in increased expression and cytoplasmic translocation of Nur77 in lung tissue in ARDS rats, and pretreatment with CsnB enhanced it (Fig. 6*A*). C-DIM8 did not change Nur77 expression in lung in ARDS rats (Fig. 6*A*). LPS induced ET-1 expression in lung tissue in ARDS rats, and pretreatment with CsnB decreased LPS-induced ET-1 expression. Pretreatment with SC-514 and SB-203580 showed similar effects. However, C-DIM8 failed to regulate ET-1 expression in lung in ARDS rats (Fig. 6*B*).

#### CsnB exerts a lung-protective effect against LPS-induced lung injury in ARDS rats.

**Fig. 7.**
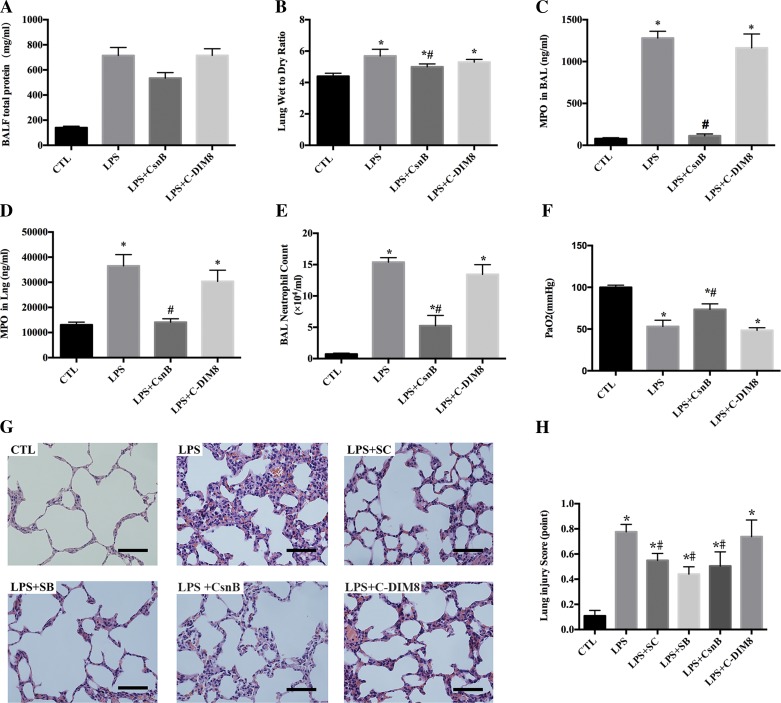
Pretreatment with CsnB attenuated lung injury in LPS-induced ARDS rats. Lung tissue and blood samples were obtained at 24 h after LPS administration. Bronchoalveolar lavage (BAL) fluid was collected at 6 h after LPS treatment. Pretreatment with CsnB attenuated LPS-induced alternation of the alveolar capillary barrier, which was measured by increased total BAL protein concentration (*A*) and increased lung wet-to-dry weight ratios (*B*). Pretreatment with CsnB inhibited LPS-induced inflammatory response, which was measured by increased BAL (*C*) and lung myeloperoxidase (MPO) concentration (*D*) and increased absolute number of neutrophils in BAL fluid (*E*). Pretreatment with CsnB alleviated LPS-induced physiological dysfunction, which was determined by hypoxemia (Pa_O_2__ <60 mmHg) (*F*). Representative hematoxylin and eosin (HE) staining images of lung section (original magnification ×400) taken from 6 rats/group. Bars, 50 μm (*G*). Lung injury was evaluated based on the histology scoring system of the American Thoracic Society workshop on experimental acute lung injury in animals. A total of 6 rats were used for each group in 2 separate experiments. Data are represented in histograms as means ± SE. *P < 0.05 vs. CTL. #*P* < 0.05 vs. LPS. The control group demonstrated a clear lung structure. The LPS group presented typical ARDS pathological manifestations: inflammatory cell infiltration, lung edema, hemorrhage, atelectasis, alveolar damage, pleural effusion, an accumulation of alveolar exudate and pulmonary interstitial thickening, but no obvious hyaline membrane formation. Pretreatment with CsnB attenuated LPS-induced lung injury, as was evident from the total lung injury score (0.78 ± 0.06 vs. 0.50 ± 0.11, *P* < 0.05). SC-514 and SB-203580 also exhibited lung-protective effects in LPS-induced ARDS rats. C-DIM8 failed to show lung-protective effects in LPS-induced ARDS rats (*H*).

Rats administered with LPS exhibited altered alveolar capillary barrier with increased total BAL protein concentration (Fig. 7*A*) and increased lung wet-to-dry weight ratios (Fig. 7*B*), upregulated inflammatory response with increased BAL (Fig. 7*C*) and lung MPO concentration (Fig. 7*D*) and increased the absolute number of neutrophils in BALF (Fig. 7*E*), physiological dysfunction with hypoxemia (Fig. 7*F*), and histological lung damage with inflammatory cell infiltration, lung edema, hemorrhage, atelectasis, alveolar damage, pleural effusion, an accumulation of alveolar exudate, and pulmonary interstitial thickening (Fig. 7*G*) and an increased lung injury score (Fig. 7*H*). Pretreatment with Nur77 agonist CsnB protected rats from LPS-induced lung injury, whereas pretreatment with Nur77 antagonist C-DIM8 had no impacts on LPS-induced ARDS rats (Fig. 7, *A–H*). Pretreatment with SC-514 and SB-203580 also exhibited lung-protective effects with decreases of lung injury score from 0.78 ± 0.06 to 0.55 ± 0.05 and from 0.78 ± 0.06 to 0.44 ± 0.06, respectively (all *P* < 0.05).

#### CsnB alleviated LPS-induced liver and kidney injury in ARDS rats.

**Fig. 8.**
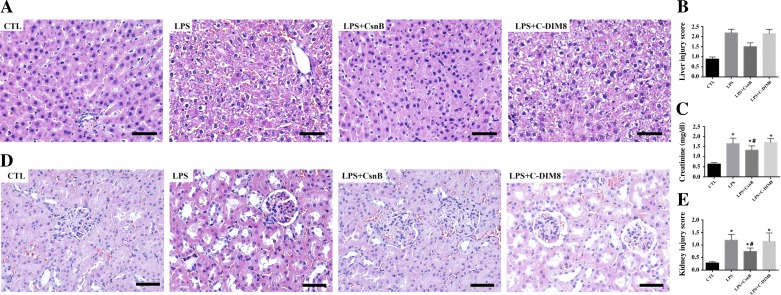
Pretreatment with CsnB attenuated kidney and liver injury in LPS-induced ARDS rats. CsnB pretreatment alleviated LPS-induced acute kidney injury and liver injury. Representative HE staining images of liver section (original magnification ×400) taken from 6 rats/group (*A*). Liver injury score (*B*). Renal dysfunction was assessed by whole blood creatinine level (*C*). *P < 0.05 vs. CTL. #*P* < 0.05 vs. LPS. Representative HE staining images of kidney section (original magnification ×400) taken from 6 rats/group (*D*). Renal pathological injury, including dilation of renal capsule cavity, edema of renal tubular epithelial cells, tubular epithelial swelling, and necrotic tubules were observed 24 h after LPS administration. Pretreatment with CsnB significantly attenuated kidney injury. Pathological scores of tubular damage (*E*). *P < 0.05 vs. CTL. #*P* < 0.05 vs. LPS. All data are expressed as means ± SE of 6 rats/group in 2 separate experiments. In the LPS + CsnB group, rats were pretreated with CsnB 1 h before LPS injection. Kidney samples were harvested at 24 h after LPS administration.

Liver pathology showed that treatment of LPS caused hemorrhage, focal necrotic and hepatocyte balloon and inflammation, and an increased liver injury score, which were partially alleviated by pretreatment with CsnB (Fig. 8, *A* and *B*). LPS administration caused significantly increased creatinine levels that were partially reversed by pretreatment with CsnB (Fig. 8*C*). Renal histopathology showed normal kidney tubules in the control rats (Fig. 8*D*). In LPS-induced ARDS rats, kidney damage, such as dilation of renal capsule cavity, edema of renal tubular epithelial cells, glomerular atrophy, tubular epithelial swelling, and necrotic tubule edema of renal tubular epithelial cells, was detected (Fig. 8*D*). Pretreatment with CsnB, to some degree, diminished LPS-induced renal interstitial edema, epithelial atrophy, and necrosis (Fig. 8*D*) and reduced kidney injury score from 1.19 ± 0.09 to 0.75 ± 0.05 (*P* < 0.05) (Fig. 8*E*). C-DIM8 had no effects on either liver injury, blood creatinine concentration, or histological kidney injury in LPS-induced ARDS rats (Fig. 8, *A–E*).

## DISCUSSION

ARDS is a clinical syndrome that is characterized by pulmonary edema, refractory hypoxemia, and multiple organ failure resulting from excessive inflammatory response to diverse insults (3). Nur77 has emerged as an important regulator of inflammation in various diseases. However, its role in the pathophysiology of ARDS is largely unknown.

### 

#### Nur77 expression and subcellular location after LPS stimulation.

The present study shows that LPS induced Nur77 expression and nuclear export both in A549 cells and ARDS rats. In addition, immunohistochemistry of lung tissue sections show that Nur77 is expressed in alveolar epithelial cells, airway epithelial cells, alveolar macrophages, and bronchial smooth muscle cells in the lungs. Nur77, coded by the immediate-early gene NR4A1, is synthesized immediately after the resting cells are stimulated by extracellular signals (69). The ability to sense and rapidly respond to changes in the cellular environment thus appears to be a hallmark of this subfamily of orphan nuclear receptors (55). Previous studies also observed rapidly increased Nur77 expression in dendritic cells, macrophages, monocytes, and adipocytes (70, 84, 96). However, LPS failed to induce Nur77 expression in muscle cells (32), suggesting that expression of Nur77 may be cell type and context specific.

Nur77 functions through both genomic and nongenomic actions, depending on the stimuli and its subcellular localization (68). Nur77 primarily locates in the nucleus, where it acts as a transcription factor by binding to its DNA response elements to regulate the expression of multiple target genes. However, Nur77 expression is induced in response to certain proapoptotic agents, and subsequently transfers from nucleus to cytoplasm and functions nongenomically through protein-protein interactions (68).

LPS-induced Nur77 nuclear export was detected in the present study, indicating that cytoplasmic Nur77 may predominantly contribute to the mechanism of ARDS. However, our results are different from those where incubation of primary human monocytes with LPS increased Nur77 nuclear translocation (65). There are several reasons to explain the disparity observed in these studies: different cell types, different dosage of LPS treatment, and different evaluation methods were used. In the present study, A549 cells were treated with LPS at a dose of 10 μg/ml, and Nur77 subcellular location is determined by laser scanning confocal microscopy. In a previous study, primary human monocytes were treated with LPS at a dose of 50 ng/ml, and Nur77 nuclear translocation was detected in nuclear extracts with Western blotting analysis (65). LPS has been shown to trigger the inflammatory response in a dose-dependent and cell-specific manner (11, 76), since LPS promotes cell proliferation (43) at low doses and has been shown to induce cell apoptosis at high doses (33).

#### NF-κB and p38 MAPK involvement in regulation of ET-1 by Nur77.

By interacting with NF-κB p65 and p38 MAPK, Nur77 inhibited LPS-induced NF-κB and p38 MAPK activation, resulting in downregulation of ET-1 in LPS-stimulated A549 cells and in the ARDS rat model. These findings give further evidence that nongenomic function of Nur77, such as protein-protein interactions, may contribute to the downregulation of LPS-induced ET-1 expression. Activation of NF-κB p65 and p38 MAPK is involved in the pathogenesis of ARDS through inducing proinflammatory mediators expression (30, 47, 100), one of which is ET-1.

This finding is consistent with the fact that Nur77 acted as a negative regulator of ET-1 in thrombin-stimulated vascular endothelial cells through an inhibitory interaction with the c-Jun/AP-1 pathway (73). Another study demonstrated that Nur77 directly interacts with p65 to block its binding to the κB elements, which subsequently leads to downregulation of NF-κB activity (61). However, this inhibitory effect of Nur77 is countered by p38α phosphorylation, and interrupting the interaction between Nur77 and p38 reduces LPS-induced inflammation (40). Nur77 has been shown to bind directly to the promoter region of IκBα, resulting in upregulation of IκBα, which leads to the eventual suppression of the NF-κB pathway in vascular endothelial cells in response to inflammatory stimuli (94).

#### Effects of Nur77 agonist and antagonist on ARDS rats.

We demonstrated that CsnB, an agonist of Nur77, decreased pulmonary neutrophilic inflammation and ET-1 expression and attenuated alveolar capillary barrier dysfunction, resulting in alleviation of hypoxemia and improvement of lung, liver, and kidney injury in LPS-induced ARDS rats, whereas, C-DIM8, a pharmacological inactivator of Nur77, exhibited neither influence on ET-1 regulation nor therapeutic effects in ARDS rats.

Neutrophils play a key role in the development of ARDS (86). Excessive accumulation of activated neutrophils in alveolar space, interstitium, and pulmonary microvasculature impairs the endothelial-epithelial barrier, resulting in protein-rich pulmonary edema, hypoxemia, and organ dysfunction (22). It has also been suggested that neutrophil migration can cause endothelial barrier mechanical destruction (95). In addition, neutrophils release proteinases, such as elastase and matrix metalloproteinases, excessive reactive mediators (31), such as cationic antimicrobial peptides, oxidants, and lipid mediators, and proinflammatory cytokines that combine to induce lung injury and extrapulmonary injury (20, 78).

ET-1 is one of the inflammatory mediators in the pathogenesis of ARDS (18, 77, 81). ET-1 induces TNF-α, IL-1, IL-6, MCP-1, and VCAM-1 production in macrophages, monocytes (3, 23, 85), mast cells (45), and human tracheal smooth muscle cells (38). In vivo, pretreatment with either ET-1 or its precursor peptide on LPS- or smoke-exposed hamsters significantly increased neutrophils in the BALF (4, 5). Elevated ET-1 levels play a systemic role in the development of multiorgan dysfunction, including lung, liver, and kidney in septic shock (17, 29). Other studies suggested that endothelin receptor antagonists, such as HJ272, significantly reduced LPS-induced inflammatory reactions, such as neutrophils in BALF, expression of tumor necrosis factor receptor 1, alveolar septal cell apoptosis, and lung histopathological injury, suggesting a potential therapeutic target for neutrophil-driven lung diseases (60).

CsnB, a pharmacological activator of Nur77 (44, 97), has been shown to exhibit anti-inflammatory effects in inflammatory bowel disease (89) and in cholesterol-induced THP-1 and U937 cells (91). Our data demonstrated therapeutic effects of CsnB against ARDS and that downregulation of ET-1 may contribute to suppression of pulmonary neutrophilic inflammation by CsnB, resulting in alleviation of endothelial-epithelial barrier dysfunction and improvement of hypoxemia and multiple organ injury.

The treatment of ARDS predominantly depends on lung-protective ventilation and judicious fluid balance, and no pharmacological therapies have demonstrated robust effectiveness (48, 72). CsnB exhibited therapeutic effects against hypoxemia and multiple organ injury, which are major causes of death in ARDS (3), indicating a potential promising target for the treatment of ARDS.

C-DIM8, an Nur77 antagonist, mimics the effects of short-interfering RNA for Nur77 and deactivates Nur77 (37, 39, 99). It has been reported that C-DIM8 is rapidly cleared from the circulation and hardly distributes in the lung when given orally (16). In this study, C-DIM8 was given through oral gavage, and this might limit the reliability of the finding that C-DIM8 has no effect on either ET-1 regulation or organ protection in ARDS rats.

Although the A549 cell line showed a functional resemblance as a type II alveolar epithelial cell, further validation using primary cells is needed.

In summary, Nur77 decreases ET-1 expression by suppressing NF-κB and p38 MAPK in LPS-stimulated A549 cells in vitro and in an LPS-induced ARDS rat model. The Nur77 agonist CsnB reduces ET-1 expression and exerts a systemic protective effect in ARDS rats.

## GRANTS

This study was supported by Grant Nos. 81160013, 81060007, and 81560321 from the National Natural Science Foundation of China and the 139 Training Plan of Guangxi Medical High Level Backbone Talents and Project of Guangxi Colleges and Universities Key Laboratory of Intensive Care Medicine and Molecular Immunology.

## DISCLOSURES

No conflicts of interest, financial or otherwise, are declared by the authors.

## AUTHOR CONTRIBUTIONS

Y.J., Y.Z., X.H., and W.L. performed experiments; Y.J., Y.Z., X.H., Y.Q., W.L., and S.X. analyzed data; Y.J., Y.Z., X.H., Y.Q., S.X., and L.P. interpreted results of experiments; Y.J., Y.Z., S.R.S., and L.P. drafted manuscript; Y.J., Y.Z., X.H., Y.Q., W.L., S.X., S.R.S., and L.P. approved final version of manuscript; X.H. and S.X. prepared figures; Y.Q. and L.P. conception and design of research; S.R.S. and L.P. edited and revised manuscript.

## References

[1] Albertine KH, Wang ZM, Michael JR Expression of endothelial nitric oxide synthase, inducible nitric oxide synthase, and endothelin-1 in lungs of subjects who died with ards. Chest 116: 101S–102S, 1999. 10424623

[2] Andey T, Patel A, Jackson T, Safe S, Singh M 1,1-Bis(3′-indolyl)-1-(p-substitutedphenyl) methane compounds inhibit lung cancer cell and tumor growth in a metastasis model. Eur J Pharm Sci 50: 227–241, 2013. 23892137 10.1016/j.ejps.2013.07.007 PMC3838903

[3] Bein T, Briegel J, Annane D Steroids are part of rescue therapy in ARDS patients with refractory hypoxemia: yes. Intensive Care Med 42: 918–920, 2016. 26883257 10.1007/s00134-015-4162-x

[4] Bellani G, Laffey JG, Pham T, Fan E, Brochard L, Esteban A, Gattinoni L, van Haren F, Larsson A, McAuley DF Epidemiology, patterns of care, and mortality for patients with acute respiratory distress syndrome in intensive care units in 50 countries. J Am Med Assoc 315: 788–800, 2016. 10.1001/jama.2016.0291 26903337

[5] Bellisai F, Morozzi G, Scaccia F, Chellini F, Simpatico A, Pecetti G, Galeazzi M Evaluation of the effect of Bosentan treatment on proinflammatory cytokine serum levels in patients affected by Systemic Sclerosis. Int J Immunopathol Pharmacol 24: 261–264, 2011. 21496413 10.1177/039463201102400134

[6] Bhavsar T, Liu XJ, Patel H, Stephani R, Cantor JO Preferential recruitment of neutrophils by endothelin-1 in acute lung inflammation induced by lipopolysaccharide or cigarette smoke. Int J Chron Obstruct Pulmon Dis 3: 477–481, 2008. 18990977 10.2147/copd.s2837 PMC2629980

[7] Bhavsar TM, Liu X, Cerreta JM, Liu M, Cantor JO Endothelin-1 potentiates smoke-induced acute lung inflammation. Exp Lung Res 34: 707–716, 2008. 19085567 10.1080/01902140802389701

[8] Blank R, Napolitano LM Epidemiology of ARDS and ALI. Crit Care Clin 27: 439–458, 2011. 21742210 10.1016/j.ccc.2011.05.005

[9] Bonta PI, van Tiel CM, Vos M, Pols TW, van Thienen JV, Ferreira V, Arkenbout EK, Seppen J, Spek CA, van der Poll T Nuclear receptors Nur77, Nurr1, and NOR-1 expressed in atherosclerotic lesion macrophages reduce lipid loading and inflammatory responses. Arterioscleros Thromb Vasc Biol 26: 2288–2288, 2006. 10.1161/01.ATV.0000238346.84458.5d 16873729

[10] Brummel N, Chandrasekhar R, Ely EW Willie Sutton and the future of acute respiratory distress syndrome research. Am J Respir Crit Care Med 191: 10–11, 2015. 25551343 10.1164/rccm.201412-2179ED PMC4299636

[11] Cario E, Rosenberg IM, Brandwein SL, Beck PL, Reinecker HC, Podolsky DK Lipopolysaccharide activates distinct signaling pathways in intestinal epithelial cell lines expressing Toll-like receptors. J Immunol 164: 966–972, 2000. 10623846 10.4049/jimmunol.164.2.966

[12] Caton PW, Nayuni NK, Murch O, Corder R Endotoxin induced hyperlactatemia and hypoglycemia is linked to decreased mitochondrial phosphoenolpyruvate carboxykinase. Life Sci 84: 738–744, 2009. 19268478 10.1016/j.lfs.2009.02.024

[13] Clerk A, Sugden PH The p38-MAPK inhibitor, SB203580, inhibits cardiac stress-activated protein kinases/c-Jun N-terminal kinases (SAPKs/JNKs). FEBS Lett 426: 93–96, 1998. 9598985 10.1016/s0014-5793(98)00324-x

[14] Comellas AP, Briva A Role of endothelin-1 in acute lung injury. Transl Res 153: 263 –271, 2009. 19446279 10.1016/j.trsl.2009.02.007 PMC3046772

[15] Comellas AP, Briva A, Dada LA, Butti ML, Trejo HE, Yshii C, Azzam ZS, Litvan J, Chen J, Lecuona E Endothelin-1 impairs alveolar epithelial function via endothelial ETB receptor. Am J Respir Crit Care Med 179: 113–122, 2009. 18948426 10.1164/rccm.200804-540OC PMC2633058

[16] De Miranda BR, Miller JA, Hansen RJ, Lunghofer PJ, Safe S, Gustafson DL, Colagiovanni D, Tjalkens RB Neuroprotective efficacy and pharmacokinetic behavior of novel anti-inflammatory para-phenyl substituted diindolylmethanes in a mouse model of Parkinson's disease. J Pharmacol Exp Ther 345: 125–138, 2013. 23318470 10.1124/jpet.112.201558 PMC6067390

[17] Deprez-Roy I, Coge F, Bertry L, Galizzi JP, Feletou M, Vanhoutte PM, Canet E Endothelin-1 pathway in human alveolar epithelial cell line A549 and human umbilical vein endothelial cells. Acta Pharmacol Sinica 21: 499–506, 2000. 11360683

[18] Fagan KA, McMurtry IF, Rodman DM Role of endothelin-1 in lung disease. Respir Res 2: 90, 2001. 11686871 10.1186/rr44 PMC59574

[19] Fenhammar J, Andersson A, Forestier J, Weitzberg E, Sollevi A, Hjelmqvist H, Frithiof R Endothelin receptor A antagonism attenuates renal medullary blood flow impairment in endotoxemic pigs. PLoS One 6: e21534, 2011. 21760895 10.1371/journal.pone.0021534 PMC3132177

[20] Fox ED, Heffernan DS, Cioffi WG, Reichner JS Neutrophils from critically ill septic patients mediate profound loss of endothelial barrier integrity. Crit Care 17: 1, 2013. 10.1186/cc13049 PMC4057230 24099563

[21] Goldie RG, D'Aprile AC, Self GJ, Rigby PJ, Henry PJ The distribution and density of receptor subtypes for endothelin-1 in peripheral lung of the rat, guinea-pig and pig. Br J Pharmacol 117: 729–735, 1996. 8646421 10.1111/j.1476-5381.1996.tb15251.x PMC1909327

[22] Grammes J, Soehnlein O Contribution of neutrophils to acute lung injury. Mol Med 17: 293, 2011. 21046059 10.2119/molmed.2010.00138 PMC3060975

[23] Hall JB, Kress JP The burden of functional recovery from ARDS. N Engl J Med 364: 1358–1359, 2011. 21470014 10.1056/NEJMe1101057

[24] Hamers AA, Vos M, Rassam F, Marinković G, Kurakula K, van Gorp PJ, de Winther MP, Gijbels MJ, de Waard V, de Vries CJ Bone marrow-specific deficiency of nuclear receptor Nur77 enhances atherosclerosis. Circ Res 110: 428–438, 2012. 22194623 10.1161/CIRCRESAHA.111.260760

[25] Han S, Mallampalli RK The acute respiratory distress syndrome: from mechanism to translation. J Immunol 194: 855–860, 2015. 25596299 10.4049/jimmunol.1402513 PMC4299926

[26] Helset E, Sildnes T, Konopski Z Endothelin-1 stimulates monocytes in vitro to release chemotactic activity identified as interleukin-8 and monocyte chemotactic protein-1. Mediat Inflamm 3: 155–160, 1994. 10.1155/S0962935194000207 PMC2367024 18472935

[27] Helset E, Sildnes T, Konopski ZS Endothelin-1 stimulates monocytes in vitro to release chemotactic activity identified as interleukin-8 and monocyte chemotactic protein-1. Mediat Inflamm 3: 155–160, 1994. 10.1155/S0962935194000207 PMC2367024 18472935

[28] Jain R, Shaul PW, Borok Z, Willis BC Endothelin-1 induces alveolar epithelial-mesenchymal transition through endothelin type A receptor-mediated production of TGF-β1. Am J Respir Cell Mol Biol 37: 38–47, 2007. 17379848 10.1165/rcmb.2006-0353OC PMC1899351

[29] Jesmin S, Yamaguchi N, Zaedi S, Nusrat Sultana S, Iwashima Y, Sawamura A, Gando S Time-dependent expression of endothelin-1 in lungs and the effects of TNF-alpha blocking peptide on acute lung injury in an endotoxemic rat model. Biomed Res 32: 9–17, 2011. 21383506 10.2220/biomedres.32.9

[30] Jing W, Chunhua M, Shumin W Effects of acteoside on lipopolysaccharide-induced inflammation in acute lung injury via regulation of NF-κB pathway in vivo and in vitro. Toxicol Appl Pharmacol 285: 128–135, 2015. 25902336 10.1016/j.taap.2015.04.004

[31] Juss JK, Herre J, Amour A, Begg M, House D, Hessel EM, Summers C, Condliffe AM, Chilvers ER Functional capacity of alveolar neutrophils in acute respiratory distress syndrome. Lancet 383: S64, 2014. 10.1016/S0140-6736(15)60370-1 26312877

[32] Kanzleiter T, Wilks D, Preston E, Ye J, Frangioudakis G, Cooney GJ Regulation of the nuclear hormone receptor nur77 in muscle: influence of exercise-activated pathways in vitro and obesity in vivo. Biochim Biophys Acta 1792: 777–782, 2009. 19447175 10.1016/j.bbadis.2009.05.002

[33] Kim KY, Shin HK, Choi JM, Hong KW Inhibition of lipopolysaccharide-induced apoptosis by cilostazol in human umbilical vein endothelial cells. J Pharmacol Exp Ther 300: 709–715, 2002. 11805237 10.1124/jpet.300.2.709

[34] Kowalczyk A, Kleniewska P, Kolodziejczyk M, Skibska B, Goraca A The role of endothelin-1 and endothelin receptor antagonists in inflammatory response and sepsis. Arch Immunol Ther Exp (Warsz) 63: 41–52, 2015. 25288367 10.1007/s00005-014-0310-1 PMC4289534

[35] Kurakula K, Koenis DS, van Tiel CM, de Vries CJ NR4A nuclear receptors are orphans but not lonesome. Biochim Biophys Acta 1843: 2543–2555, 2014. 24975497 10.1016/j.bbamcr.2014.06.010

[36] Kurakula K, Vos M, Logiantara A, Roelofs JJ, Nieuwenhuis MA, Koppelman GH, Postma DS, van Rijt LS, de Vries CJ Nuclear receptor Nur77 attenuates airway inflammation in mice by suppressing NF-κB activity in lung epithelial cells. J Immunol 195: 1388–1398, 2015. 26170382 10.4049/jimmunol.1401714

[37] Lee SO, Abdelrahim M, Yoon K, Chintharlapalli S, Papineni S, Kim K, Wang H, Safe S Inactivation of the orphan nuclear receptor TR3/Nur77 inhibits pancreatic cancer cell and tumor growth. Cancer Res 70: 6824–6836, 2010. 20660371 10.1158/0008-5472.CAN-10-1992 PMC2988472

[38] Lee SL, Wesselschmidt RL, Linette GP, Kanagawa O, Russell JH, Milbrandt J Unimpaired thymic and peripheral T cell death in mice lacking the nuclear receptor NGFI-B (Nur77). Science 269: 532–535, 1995. 7624775 10.1126/science.7624775

[39] Lee SO, Andey T, Jin UH, Kim K, Singh M, Safe S The nuclear receptor TR3 regulates mTORC1 signaling in lung cancer cells expressing wild-type p53. Oncogene 31: 3265–3276, 2012. 22081070 10.1038/onc.2011.504 PMC3299891

[40] Li L, Liu Y, Chen Hz Li Fw Wu Jf Zhang Hk He Jp Xing Yz Chen Y, Wang Wj Impeding the interaction between Nur77 and p38 reduces LPS-induced inflammation. Nat Chem Biol 11: 339–346, 2015. 25822914 10.1038/nchembio.1788

[41] Li XM, Lu XX, Xu Q, Wang JR, Zhang S, Guo PD, Li JM, Wu H Nur77 deficiency leads to systemic inflammation in elderly mice. J Inflamm 12: 40, 2015. 10.1186/s12950-015-0085-0 PMC4480882 26113803

[42] Lin CC, Lin WN, Hou WC, Hsiao LD, Yang CM Endothelin-1 induces VCAM-1 expression-mediated inflammation via receptor tyrosine kinases and Elk/p300 in human tracheal smooth muscle cells. Am J Physiol Lung Cell Mol Physiol 309: L211–L225, 2015. 26071554 10.1152/ajplung.00232.2014

[43] Lin FY, Chen YH, Chen YL, Wu TC, Li CY, Chen JW, Lin SJ Ginkgo biloba extract inhibits endotoxin-induced human aortic smooth muscle cell proliferation via suppression of toll-like receptor 4 expression and NADPH oxidase activation. J Agricul Food Chem 55: 1977–1984, 2007. 10.1021/jf062945r 17266329

[44] Liu JJ, Zeng HN, Zhang LR, Zhan YY, Chen Y, Wang Y, Wang J, Xiang SH, Liu WJ, Wang WJ A unique pharmacophore for activation of the nuclear orphan receptor Nur77 in vivo and in vitro. Cancer Res 70: 3628–3637, 2010. 20388790 10.1158/0008-5472.CAN-09-3160

[45] Liu Q, Wu H, Chim SM, Zhou L, Zhao J, Feng H, Wei Q, Wang Q, Zheng MH, Tan RX SC-514, a selective inhibitor of IKKβ attenuates RANKL-induced osteoclastogenesis and NF-κB activation. Biochem Pharmacol 86: 1775–1783, 2013. 24091016 10.1016/j.bcp.2013.09.017

[46] Ma JQ, Li Z, Xie WR, Liu CM, Liu SS Quercetin protects mouse liver against CCl 4-induced inflammation by the TLR2/4 and MAPK/NF-κB pathway. Int Immunopharmacol 28: 531–539, 2015. 26218279 10.1016/j.intimp.2015.06.036

[47] Ma MM, Li Y, Liu XY, Zhu WW, Ren X, Kong GQ, Huang X, Wang LP, Luo LQ, Wang XZ Cyanidin-3-O-glucoside ameliorates lipopolysaccharide-induced injury both in vivo and in vitro suppression of NF-κB and MAPK pathways. Inflammation 38: 1669–1682, 2015. 25752620 10.1007/s10753-015-0144-y

[48] Mac Sweeney R, McAuley DF Acute respiratory distress syndrome. Lancet In press. 10.1016/S0140-6736(16)00578-X PMC7138018 27133972

[49] Markewitz BA, Kohan DE, Michael JR Endothelin-1 synthesis, receptors, and signal transduction in alveolar epithelium: evidence for an autocrine role. Am J Physiol Lung Cell Mol Physiol 268: L192–L200, 1995. 10.1152/ajplung.1995.268.2.L192 7864140

[50] Matsushima H, Yamada N, Matsue H, Shimada S The effects of endothelin-1 on degranulation, cytokine, and growth factor production by skin-derived mast cells. Eur J Immunol 34: 1910–1919, 2004. 15214039 10.1002/eji.200424912

[51] Matthay MA, Ware LB Resolution of alveolar edema in acute respiratory distress syndrome. Physiology and biology. Am J Respir Crit Care Med 192: 124–125, 2015. 26177166 10.1164/rccm.201505-0938ED

[52] Matthay MA, Ware LB, Zimmerman GA The acute respiratory distress syndrome. J Clin Invest 122: 2731–2740, 2012. 22850883 10.1172/JCI60331 PMC3408735

[53] Matute-Bello G, Downey G, Moore BB, Groshong SD, Matthay MA, Slutsky AS, Kuebler WM An official American Thoracic Society workshop report: features and measurements of experimental acute lung injury in animals. Am J Respir Cell Mol Biol 44: 725–738, 2011. 21531958 10.1165/rcmb.2009-0210ST PMC7328339

[54] Matute-Bello G, Frevert CW, Martin TR Animal models of acute lung injury. Am J Physiol Lung Cell Mol Physiol 295: L379–L399, 2008. 18621912 10.1152/ajplung.00010.2008 PMC2536793

[55] Maxwell MA, Muscat GE The NR4A subgroup: immediate early response genes with pleiotropic physiological roles. Nucl Recept Signal 4: e002, 2006. 16604165 10.1621/nrs.04002 PMC1402209

[56] McKenna S, Gossling M, Bugarini A, Hill E, Anderson AL, Rancourt RC, Balasubramaniyan N, El Kasmi KC, Wright CJ Endotoxemia induces IκBβ/NF-κB-dependent endothelin-1 expression in hepatic macrophages. J Immunol 195: 3866–3879, 2015. 26342031 10.4049/jimmunol.1501017 PMC4730915

[57] McMorrow J, Murphy E Inflammation: a role for NR4A orphan nuclear receptors? Biochem Soc Trans 39: 688, 2011. 21428963 10.1042/BST0390688

[58] Michael JR, Markewitz BA Endothelins and the lung. Am J Respir Crit Care Med 154: 555–581, 1996. 8810589 10.1164/ajrccm.154.3.8810589

[59] Miller SI, Wallace RJ Jr, Musher DM, Septimus EJ, Kohl S, Baughn RE Hypoglycemia as a manifestation of sepsis. Am J Med 68: 649–654, 1980. 6990758 10.1016/0002-9343(80)90250-8

[60] Munford RS Endotoxemia-menace, marker, or mistake? J Leukocyte Biol 100: 687–698, 2016. 27418356 10.1189/jlb.3RU0316-151R PMC5014740

[61] Murphy EP, Crean D Molecular interactions between NR4A orphan nuclear receptors and NF-κB are required for appropriate inflammatory responses and immune cell homeostasis. Biomolecules 5: 1302–1318, 2015. 26131976 10.3390/biom5031302 PMC4598753

[62] Nakano Y, Tasaka S, Saito F, Yamada W, Shiraishi Y, Ogawa Y, Koh H, Hasegawa N, Fujishima S, Hashimoto S Endothelin-1 level in epithelial lining fluid of patients with acute respiratory distress syndrome. Respirology 12: 740–743, 2007. 17875064 10.1111/j.1440-1843.2007.01115.x

[63] Neff SB, Z'Graggen BR, Neff TA, Jamnicki-Abegg M, Suter D, Schimmer RC, Booy C, Joch H, Pasch T, Ward PA, Beck-Schimmer B Inflammatory response of tracheobronchial epithelial cells to endotoxin. Am J Physiol Lung Cell Mol Physiol 290: L86–L96, 2006. 16100285 10.1152/ajplung.00391.2004

[64] Oslund KL, Hyde DM, Putney LF, Alfaro MF, Walby WF, Tyler NK, Schelegle ES Activation of neurokinin-1 receptors during ozone inhalation contributes to epithelial injury and repair. Am J Respir Cell Mol Biol 39: 279–288, 2008. 18390473 10.1165/rcmb.2008-0009OC PMC2542446

[65] Pang T, Wang J, Benicky J, Saavedra JM Minocycline ameliorates LPS-induced inflammation in human monocytes by novel mechanisms including LOX-1, Nur77 and LITAF inhibition. Biochim Biophys Acta 1820: 503–510, 2012. 22306153 10.1016/j.bbagen.2012.01.011 PMC3307860

[66] Patel S, Liu X, Liu M, Stephani R, Patel H, Cantor J HJP272, a novel endothelin receptor antagonist, attenuates lipopolysaccharide-induced acute lung injury in hamsters. Lung 192: 803–810, 2014. 25087133 10.1007/s00408-014-9628-z

[67] Patel S, Liu X, Liu M, Stephani R, Patel H, Cantor J HJP272, a novel endothelin receptor antagonist, attenuates lipopolysaccharide-induced acute lung injury in hamsters. Lung 192: 803–810, 2014. 25087133 10.1007/s00408-014-9628-z

[68] Pawlak A, Strzadala L, Kalas W Non-genomic effects of the NR4A1/Nur77/TR3/NGFIB orphan nuclear receptor. Steroids 95: 1–6, 2015. 25555471 10.1016/j.steroids.2014.12.020

[69] Pearen MA, Muscat GE Minireview: Nuclear hormone receptor 4A signaling: implications for metabolic disease. Mol Endocrinol 24: 1891–1903, 2010. 20392876 10.1210/me.2010-0015 PMC5417389

[70] Pei L, Castrillo A, Chen M, Hoffmann A, Tontonoz P Induction of NR4A orphan nuclear receptor expression in macrophages in response to inflammatory stimuli. J Biol Chem 280: 29256–29262, 2005. 15964844 10.1074/jbc.M502606200

[71] Pei L, Castrillo A, Tontonoz P Regulation of macrophage inflammatory gene expression by the orphan nuclear receptor Nur77. Mol Endocrinol 20: 786–794, 2006. 16339277 10.1210/me.2005-0331

[72] Pipeling MR, Fan E Therapies for refractory hypoxemia in acute respiratory distress syndrome. J Am Med Assoc 304: 2521–2527, 2010. 10.1001/jama.2010.1752 21139113

[73] Qin Q, Chen M, Yi B, You X, Yang P, Sun J Orphan nuclear receptor Nur77 is a novel negative regulator of endothelin-1 expression in vascular endothelial cells. J Mol Cell Cardiol 77: 20–28, 2014. 25284689 10.1016/j.yjmcc.2014.09.027 PMC4312239

[74] Raetzsch CF, Brooks NL, Alderman JM, Moore KS, Hosick PA, Klebanov S, Akira S, Bear JE, Baldwin AS, Mackman N, Combs TP Lipopolysaccharide inhibition of glucose production through the Toll-like receptor-4, myeloid differentiation factor 88, and nuclear factor kappa b pathway. Hepatology 50: 592–600, 2009. 19492426 10.1002/hep.22999 PMC2822400

[75] Riviello ED, Kiviri W, Twagirumugabe T, Mueller A, Banner-Goodspeed VM, Officer L, Novack V, Mutumwinka M, Talmor DS, Fowler RA Hospital incidence and outcomes of the acute respiratory distress syndrome using the kigali modification of the berlin definition. Am J Respir Crit Care Med 193: 52–59, 2016. 26352116 10.1164/rccm.201503-0584OC

[76] Schulz C, Farkas L, Wolf K, Krätzel K, Eissner G, Pfeifer M Differences in LPS-induced activation of bronchial epithelial cells (BEAS-2B) and type ii-like pneumocytes (A-549). Scand J Immunol 56: 294–302, 2002. 12193231 10.1046/j.1365-3083.2002.01137.x

[77] Sessa WC, Kaw S, Hecker M, Vane JR The biosynthesis of endothelin-1 by human polymorphonuclear leukocytes. Biochem Biophys Res Commun 174: 613–618, 1991. 1993057 10.1016/0006-291x(91)91461-k

[78] Summers C, Singh NR, White JF, Mackenzie IM, Johnston A, Solanki C, Balan K, Peters AM, Chilvers ER Pulmonary retention of primed neutrophils: a novel protective host response, which is impaired in the acute respiratory distress syndrome. Thorax 69: 623–629, 2014. 24706039 10.1136/thoraxjnl-2013-204742 PMC4055272

[79] Sweeney RM, McAuley DF Acute respiratory distress syndrome. Lancet In press. 10.1016/S0140-6736(16)00578-X PMC7138018 27133972

[80] Takeuchi O, Hoshino K, Kawai T, Sanjo H, Takada H, Ogawa T, Takeda K, Akira S Differential roles of TLR2 and TLR4 in recognition of gram-negative and gram-positive bacterial cell wall components. Immunity 11: 443–451, 1999. 10549626 10.1016/s1074-7613(00)80119-3

[81] Teder P, Noble PW A cytokine reborn? Endothelin-1 in pulmonary inflammation and fibrosis. Am J Respir Cell Mol Biol 23: 7–10, 2000. 10873147 10.1165/ajrcmb.23.1.f192

[82] Villar J, Blanco J, Anon JM, Santos-Bouza A, Blanch L, Ambros A, Gandia F, Carriedo D, Mosteiro F, Basaldua S, Fernandez RL, Kacmarek RM, Network A The ALIEN study: incidence and outcome of acute respiratory distress syndrome in the era of lung protective ventilation. Intensive Care Med 37: 1932–1941, 2011. 21997128 10.1007/s00134-011-2380-4

[83] Villar J, Sulemanji D, Kacmarek RM The acute respiratory distress syndrome: incidence and mortality, has it changed? Curr Opin Crit Care 20: 3–9, 2014. 24309954 10.1097/MCC.0000000000000057

[84] Wang SCM, Myers SA, Eriksson NA, Fitzsimmons RL, Muscat GE Nr4a1 siRNA expression attenuates α-MSH regulated gene expression in 3T3–L1 adipocytes. Mol Endocrinol 25: 291–306, 2011. 21239615 10.1210/me.2010-0231 PMC5417310

[85] Wheeler AP, Bernard GR Acute lung injury and the acute respiratory distress syndrome: a clinical review. Lancet 369: 1553–1564, 2007. 17482987 10.1016/S0140-6736(07)60604-7

[86] Williams AE, Chambers RC The mercurial nature of neutrophils: still an enigma in ARDS? Am J Physiol Lung Cell Mol Physiol 306: L217–L230, 2014. 24318116 10.1152/ajplung.00311.2013 PMC3920201

[87] Winoto A, Littman DR Nuclear hormone receptors in T lymphocytes. Cell 109: S57–S66, 2002. 11983153 10.1016/s0092-8674(02)00710-9

[88] Woronicz JD, Calnan B, Ngo V, Winoto A Requirement for the orphan steroid receptor Nur77 in apoptosis of T-cell hybridomas. Nature 367: 277–281, 1994. 8121493 10.1038/367277a0

[89] Wu H, Li XM, Wang JR, Gan WJ, Jiang FQ, Liu Y, Zhang XD, He XS, Zhao YY, Lu XX NUR77 exerts a protective effect against inflammatory bowel disease by negatively regulating the TRAF6/TLR-IL-1R signalling axis. J Pathol 238: 457–469, 2016. 26564988 10.1002/path.4670

[90] Xiang S, Zeng Y, Xiong B, Qin Y, Huang X, Jiang Y, Luo W, Sooranna SR, Pinhu L Transforming growth factor beta 1 induced endothelin-1 release is peroxisome proliferator-activated receptor gamma dependent in A549 cells. J Inflamm (Lond) 13: 19, 2016. 27293383 10.1186/s12950-016-0128-1 PMC4902962

[91] Xie X, Song X, Yuan S, Cai H, Chen Y, Chang X, Liang B, Huang D Histone acetylation regulates orphan nuclear receptor NR4A1 expression in hypercholesterolaemia. Clin Sci 129: 1151–1161, 2015. 26396259 10.1042/CS20150346

[92] Yanagisawa M, Kurihara H, Kimura S, Tomobe Y, Kobayashi M, Mitsui Y, Yazaki Y, Goto K, Masaki T A novel potent vasoconstrictor peptide produced by vascular endothelial cells. Nature 332: 411–415, 1988. 2451132 10.1038/332411a0

[93] Yeager ME, Belchenko DD, Nguyen CM, Colvin KL, Ivy DD, Stenmark KR Endothelin-1, the unfolded protein response, and persistent inflammation: role of pulmonary artery smooth muscle cells. Am J Respir Cell Mol Biol 46: 14–22, 2012. 21778413 10.1165/rcmb.2010-0506OC PMC3262656

[94] You B, Jiang YY, Chen S, Yan G, Sun J The orphan nuclear receptor Nur77 suppresses endothelial cell activation through induction of IκBα expression. Circ Res 104: 742–749, 2009. 19213954 10.1161/CIRCRESAHA.108.192286

[95] Yuan SY, Shen Q, Rigor RR, Wu MH Neutrophil transmigration, focal adhesion kinase and endothelial barrier function. Microvasc Res 83: 82–88, 2012. 21864543 10.1016/j.mvr.2011.06.015 PMC3232323

[96] Zanoni I, Ostuni R, Capuano G, Collini M, Caccia M, Ronchi AE, Rocchetti M, Mingozzi F, Foti M, Chirico G CD14 regulates the dendritic cell life cycle after LPS exposure through NFAT activation. Nature 460: 264–268, 2009. 19525933 10.1038/nature08118

[97] Zhan Y, Du X, Chen H, Liu J, Zhao B, Huang D, Li G, Xu Q, Zhang M, Weimer BC Cytosporone B is an agonist for nuclear orphan receptor Nur77. Nat Chem Biol 4: 548–556, 2008. 18690216 10.1038/nchembio.106

[98] Zhang Z, Jian X, Zhang W, Wang J, Zhou Q Using bosentan to treat paraquat poisoning-induced acute lung injury in rats. PloS One 8: e75943, 2013. 24155875 10.1371/journal.pone.0075943 PMC3796527

[99] Zhao BX, Chen HZ, Lei NZ, Li GD, Zhao WX, Zhan YY, Liu B, Lin SC, Wu Q p53 mediates the negative regulation of MDM2 by orphan receptor TR3. EMBO J 25: 5703–5715, 2006. 17139261 10.1038/sj.emboj.7601435 PMC1698882

[100] Zhou E, Li Y, Wei Z, Fu Y, Lei H, Zhang N, Yang Z, Xie G Schisantherin A protects lipopolysaccharide-induced acute respiratory distress syndrome in mice through inhibiting NF-κB and MAPKs signaling pathways. Int Immunopharmacol 22: 133–140, 2014. 24975658 10.1016/j.intimp.2014.06.004

